# A computer simulation model of Wolbachia invasion for disease vector population modification

**DOI:** 10.1186/s12859-015-0746-2

**Published:** 2015-10-05

**Authors:** Mauricio Guevara-Souza, Edgar E. Vallejo

**Affiliations:** Department of Computer Science, Tecnologico de Monterrey, Carretera a lago de Guadalupe km 3.5, Col. Margarita Maza de Juarez, Atizapan de Zaragoza, Adolfo Lopez Mateos, 52926 Mexico

**Keywords:** Dengue, Wolbachia infection, Computer simulation

## Abstract

**Background:**

*Wolbachia* invasion has been proved to be a promising alternative for controlling vector-borne diseases, particularly Dengue fever. Creating computer models that can provide insight into how vector population modification can be achieved under different conditions would be most valuable for assessing the efficacy of control strategies for this disease.

**Methods:**

In this paper, we present a computer model that simulates the behavior of native mosquito populations after the introduction of mosquitoes infected with the *Wolbachia* bacteria. We studied how different factors such as fecundity, fitness cost of infection, migration rates, number of populations, population size, and number of introduced infected mosquitoes affect the spread of the *Wolbachia* bacteria among native mosquito populations.

**Results:**

Two main scenarios of the island model are presented in this paper, with infected mosquitoes introduced into the largest source population and peripheral populations. Overall, the results are promising; *Wolbachia* infection spreads among native populations and the computer model is capable of reproducing the results obtained by mathematical models and field experiments.

**Conclusions:**

Computer models can be very useful for gaining insight into how *Wolbachia* invasion works and are a promising alternative for complementing experimental and mathematical approaches for vector-borne disease control.

## Background

Dengue is considered the most rapidly spreading viral disease in the world. There are 30 more human infections nowadays than 50 years ago. The World Health Organization estimates that over 2.5 billion people in more than 100 countries are at risk of infection, with Southeast Asia, the Americas, and Western Pacific as the most vulnerable areas [1].

Infection rates fluctuate between 50 and 100 million infected people each year, 500,000 of whom develop hemorrhagic fever that causes up to 25,000 deaths annually worldwide [1]. More recent forecasts estimate 390 million Dengue infections per year with approximately 96 million suffering from serious symptoms of the disease [1].

The economic burden of medical treatment for infected people and the loss of productivity in countries where Dengue is endemic are very high. The cost of Dengue fever has been estimated to be approximately 2.1 billion dollars per year in the Americas alone, with the cost increasing from year to year. Dengue is an illness that consumes more resources from the health systems of affected countries than any other disease [2].

Dengue is a human virus transmitted from individual to individual by the *Aedes aegypti* mosquito, a species that is commonly found in the workplace and homes in tropical areas. Dengue viruses can be grouped into four serotypes, all of which are capable of producing Dengue fever and Dengue hemorrhagic fever. It is believed that once a serotype of Dengue is acquired, the risk of developing hemorrhagic Dengue increases [1].

Under the circumstances, it is clear that creating new ways to reduce Dengue incidence is imperative. Currently, there are no effective treatments against Dengue. Several drugs can be used to alleviate the symptoms and help the body resist the illness, but there is no specific drug that kills the pathogen [3]. Although there is a vaccine for Dengue, it is not currently available for commercial production [1].

The introduction of modified mosquitoes into wild populations is a disease control strategy that seems promising in principle [4, 5]. The invasion of disease-carrying populations by mosquitoes that are refractory to the disease is a convenient approach that could be more effective than traditional vector control strategies such as the use of insecticide, and hopefully less harmful to the environment.

One feasible alternative that seems worth exploring is the introduction of mosquitoes infected with the *Wolbachia* bacteria into wild populations for Dengue disease control. *Wolbachia* bacteria produces a series of modifications in the reproduction mechanism of its host, such as cytoplasmic incompatibility, that can contribute to the establishment of immune populations [6].

Furthermore, in other species of insects the *Wolbachia* bacteria has been observed to provide virus resistance to its hosts and immunity to some diseases. However, it has previously been suggested that *Wolbachia* infection produces a loss of fitness in its hosts [5]. Conversely, previous experiments have demonstrated that in some *Wolbachia* strains, the cytoplasmic incompatibility driver can be capable of overcoming the loss of fitness in the infected hosts and imperfect maternal transmission [7]. In principle, these conditions should promote the rapid invasion of the host population [5].

Currently, the ongoing project “Eliminate Dengue” aims to develop a natural approach to control Dengue using *Aedes aegypti* mosquitoes infected with the *Wolbachia* bacteria. To date, there have been releases of infected mosquitoes in Australia with field data demonstrating seemingly promising results. However, it is premature to conclude to what extent this approach will work [8]. To monitor *Wolbachia* invasion in native mosquito populations, a collection of mosquitoes are captured to test if they are infected. Collecting individuals in the field can be onerous, particularly during the dry season when only a few individuals are likely to be captured making the measures statistically unreliable [5]. In coming years, several releases are planned in Brazil, China, Vietnam, and Indonesia [8].

Theoretical models on the dynamics of *Wolbachia* invasion have previously been developed [9, 10]. In principle, these models should be able to explain the dynamics of an invasion of native populations [11]. However, mathematical population dynamics models are often based on strong assumptions such as unbounded population sizes, probability calculations that are difficult in practice, and random mating. Another disadvantage of mathematical models is their intrinsic complexity [12].

The majority of these models require a solid mathematical background to understand and use them as predictive tools. We believe that using computer simulation models could be more user-friendly than using mathematical models for making predictions on *Wolbachia* invasion. Additionally, the environment in population dynamics tends to change over time and mathematical models do not adapt well to changes, causing a loss of accuracy in the predictions [12]. In this context, we believe that computational simulations can be very useful tools complementing mathematical models and in some cases, capable of producing similar predictions. We believe that the results obtained by computer models could be useful in planning and carrying out successful experimental work.

Previously, we have developed computational tools and models as part of our research program, the aim of which is to use computer models to provide insight into the population dynamics of disease vectors. We have simulated a variety of gene drive mechanisms, such as transposable elements and the maternal effect dominant embryonic arrest, to predict the effectiveness and feasibility of these population replacement strategies for vector-borne disease control [13–16].

In this paper, we present a series of simulations on *Wolbachia* invasion of simulated vector populations. A variety of scenarios are explored using different variables. The main objective of this work is to formulate a computational model that could be useful in determining the conditions required by *Wolbachia* to invade a native population.

The computational model proposed in this study could be used to provide valuable insight into the conditions required for *Wolbachia* invasion to occur, including the proportion of *Wolbachia*-infected individuals needed to realize invasion of a native mosquito population, among others. We present experimental results to show the usefulness of the proposed model.

### *Wolbachia* bacteria

*Wolbachia pipientis* is a type of bacteria that infects a wide variety of invertebrates. It is estimated that approximately 70 % of insect species are infected with this bacteria [17].

The bacteria can spread rapidly in an uninfected population owing to the cytoplasmic incompatibility mechanism that is induced in its hosts [18, 19]. This mechanism causes the progeny of a female that is not infected with *Wolbachia* and a male that is infected to die by reducing egg hatch. If the female is infected, the offspring will survive and will be infected with *Wolbachia* irrespective of the infection status of the male. Previous studies have also suggested a decrease in fitness and fecundity of the infected hosts [5, 7].

There are several strains of *Wolbachia* bacteria that infect insects in nature, but two strains in particular have been proposed for use in population modification [8].

The first strain, called *wMelPop*, shortens the life span of infected mosquitoes by approximately half. The Dengue infection cycle takes about 12 days to complete, starting when the mosquito bites an infected person and ending when the virus can be transmitted by the mosquito to another person. During this time, the virus replicates inside the mosquito until it reaches the salivary glands. When the mosquito takes a blood meal, the infected saliva enters the host causing a new infection [1]. Because only old mosquitoes can transmit Dengue or malaria owing to the long infection cycles of these diseases, this strain could be used as a disease control. This strain has, however, only been tested in laboratory experiments [8].

A second strain of *Wolbachia*, called *wMel*, provides some virus resistance to its hosts. This mechanism could result in the rapid invasion of the host population and, therefore, in a promising disease control mechanism as virus resistance prevents the mosquitoes from being infected with the Dengue pathogen [8]. However, there is a decrease in fitness in the infected individuals preventing the bacteria from spreading, although it is believed that the cytoplasmic incompatibility that *wMel* induces in its hosts is strong enough to negate the fitness cost [5].

Biologists have been trying to infect mosquito eggs with *Wolbachia* for several years with poor results. More recently, however, using micro injection they have been able to infect *Aedes aegypti* eggs with a strain of *Wolbachia* from the fruit fly [20].

Using this important discovery, examining scenarios and mechanisms that can lead to *Wolbachia* invasion of a wild population will be valuable for studying the potential of biological control strategies for this disease.

## Methods

The availability of data in the literature on mosquito population structure and distribution, population sizes, and migration rates, is not abundant. The experiments presented in this section are based on examples found in the literature and information gathered through personal communication with biologists [5, 7].

**Fig. 1:**
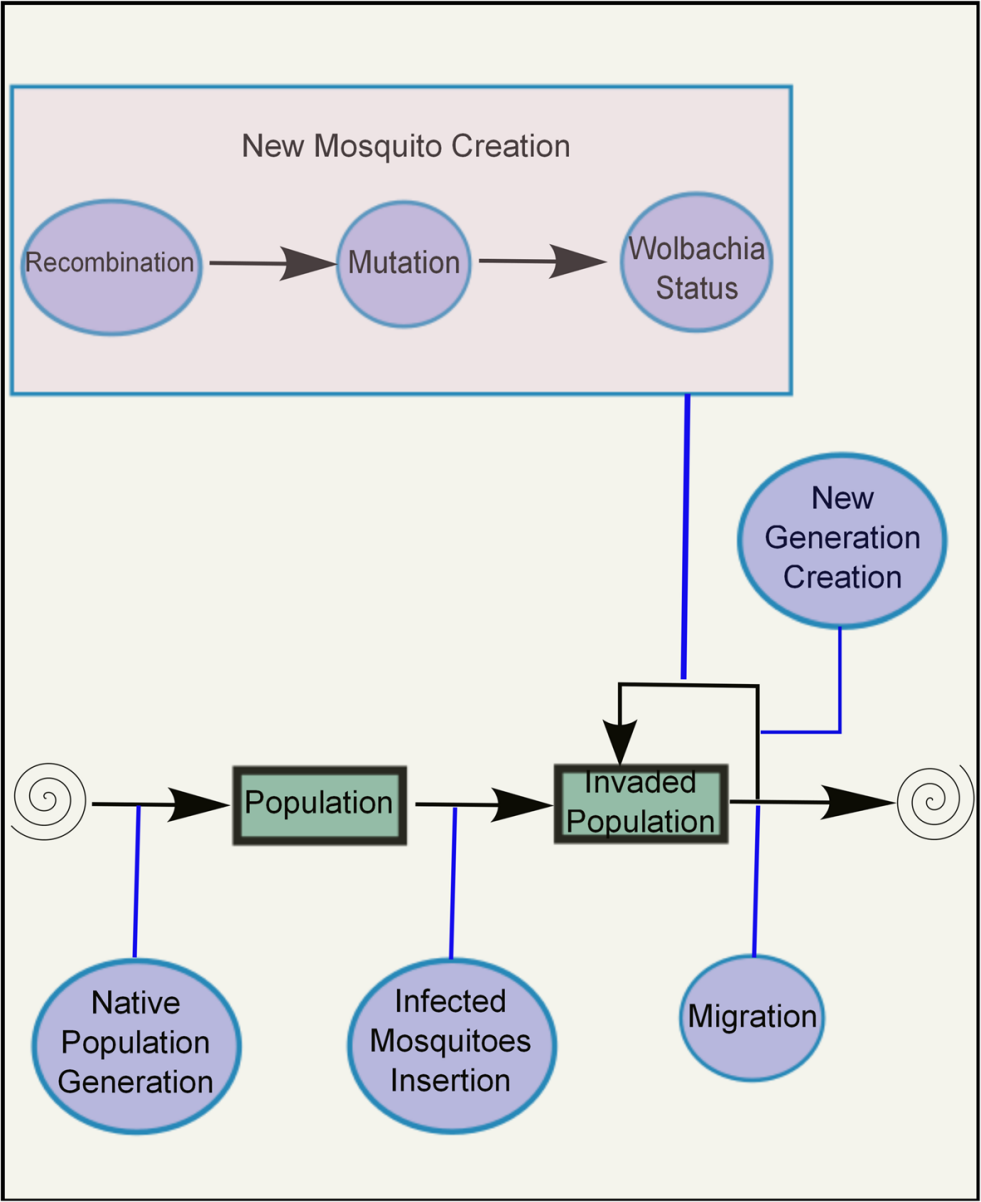
Computer model used to run the experiments

The computer model used for our experiments consists of a collection of mosquito populations that are connected via migration (see Fig. 1). In nature, it is common for mosquito populations to be spread over a geographic location, generally near villages and water bodies, with some individual exchange between them [21]. Although migration is an important factor for introducing genetic diversity in populations [22], it has a downside. According to mathematical models, it tends to decrease the *Wolbachia* invasion rate [9].

In the proposed computational model, we used a parallel algorithm and where possible, each population was simulated on an independent CPU core isolated from the other populations. We used the cytoplasmic incompatibility mechanism to alter the reproduction process of the population to observe if *Wolbachia* is capable of invading a native population.

### Mosquito representation

For each mosquito in the population, we modeled a set of attributes representing what we considered the most important features for modeling *Wolbachia* invasion. The first property of the mosquito is its chromosome represented by an array of letters, each of which belongs to the DNA alphabet (A,G,C,T).

It is important to note that *Wolbachia* infection does not alter the DNA of the host; we included the chromosome of the mosquito to provide data for use in conducting evolutionary analyses such as phylogenetic reconstruction, as a future work. The chromosome was kept small to save some computer resources as chromosome length does not alter the results of the simulations, but was long enough to allow genetic traces of the individuals to be tracked. The second feature we included in the model was gender. Gender differentiation between individuals is important in the reproduction process. The gender attribute can have only two values, male and female.

Another important component of our model is explicit representation of geographic location using a square population grid. For this purpose, we used a pair of variables to track the column and row of every mosquito. Location within the population is important particularly in the reproduction stage because mosquitoes mate with other mosquitoes in their surroundings.

A Boolean flag was used to record whether an individual was infected with *Wolbachia*, to simulate the invasion process.

Finally, we kept track of the fitness of an individual, including the fitness cost associated with *Wolbachia* infection [5]. Fitness is probably the most important characteristic of an individual because it controls its reproductive capability. A high fitness value increases the probability of an individual to generate offspring and also dictates the number of offspring it will produce. The fitness of the initial population is assigned randomly between 0 and 100. The fitness of the offspring is calculated as the average of the fitness values of the parents with a small perturbation that increases or decreases the fitness slightly.

Fitness is a very abstract concept that depends not only on genetic factors, but also on the environment. Measuring the fitness of an individual is very complicated because it is an intrinsic and idealized value. We used a numerical scale to identify simply how well the individuals had adapted to the environment [23].

### Population structure

**Fig. 2:**
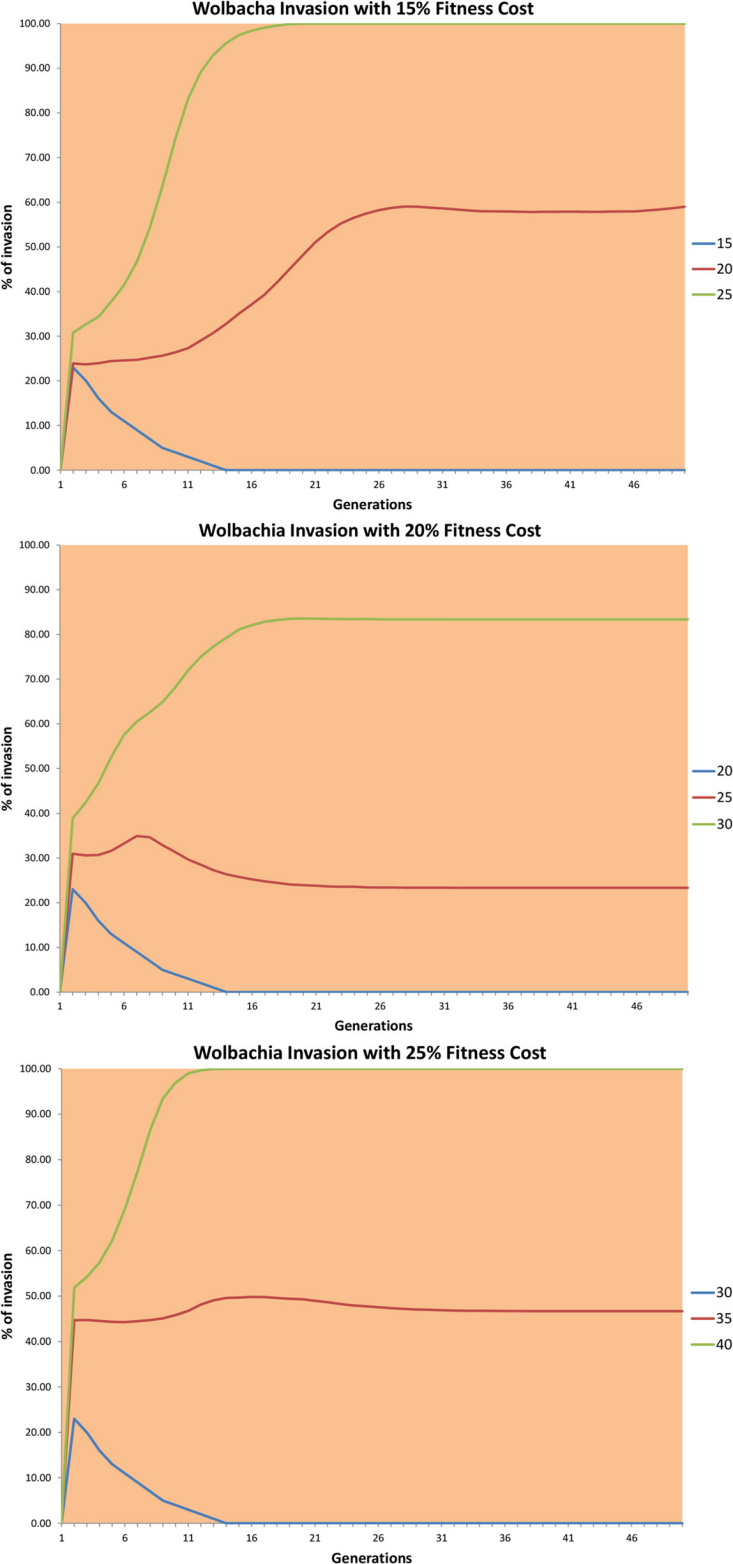
Population structure. Individuals in blue squares indicate possible mates for the female in the green square representing the center of the neighborhood. Individuals in red are infected with *Wolbachia*

The habitat of the mosquitoes is simulated using a two-dimensional toroidal grid. The location of each individual in the grid is important in the reproduction stage because we limited females from pairing only with males occurring within their neighborhood (see Fig. 2).

The population is composed of approximately half males and half females. The gender of the offspring is determined randomly at the time of breeding, and thus, this composition can vary slightly from generation to generation. The size of the population is fixed for the entire simulation.

In nature, the density of the population of mosquitoes varies significantly throughout the year because it depends heavily on climatic factors such as rain and temperature. As *Aedes aegypti* mosquitoes prefer artificial water containers as breeding sites, they do not rely entirely on climatic factors. This means that if there are sufficient artificial water supplies to breed, the *Aedes aegypti* population remains almost the same throughout the year [24]. This is the reason why we fixed the size of the mosquito populations in our simulations.

We used the proposed population grid to simulate as closely as possible, how reproduction takes place in natural populations. It is known that the location of an individual is not random, but depends on the fitness of the individual. In many populations, individuals with the highest fitness tend to gather together in the center of the population, while the less adapted ones are relegated to remote locations [25].

### *Wolbachia* infection

In our experiments, *Wolbachia* infection produces unidirectional cytoplasmic incompatibility in its hosts and a decrease in fitness of the infected individual. The infection process occurs at the beginning of the simulation. Populations of mosquitoes evolve independently, with some infected with *Wolbachia* and other native populations remaining uninfected. When the simulation starts, the populations are combined to observe the interaction between uninfected and infected populations.

The fitness decrease due to *Wolbachia* infection, the number of populations simulated, where infected mosquitoes are introduced, and the percentage of infected mosquitoes introduced, vary from experiment to experiment. At the end of each experiment, we calculated the rate of *Wolbachia* invasion in each population.

### Mating restriction

In mathematical models and ideal populations, reproduction relies on random mating [26]. However, very few populations exhibit random mating in the wild. In nature, there are a variety of circumstances that make random mating impossible, especially geographic constraints. In our computer model, we simulated a geographic restriction to mimic more faithfully how reproduction occurs in native populations.

### Genetic operators

#### Selection

Random mating was not used to choose the parents in the reproduction process owing to the incorporation of two important restrictions. The first significant restriction is the gender of the individuals. First, we randomly selected the female from the population and then we found a suitable male.

In the second restriction, the males that can mate with a selected female are only those that exist within the neighborhood of the female.

To emulate the effect of fitness on reproduction, we used a selection mechanism for choosing both male and female. This mechanism is similar to the tournament selection used in evolutionary algorithms that involves randomly selecting a collection of individuals from the population and then, based on the fitness values, choosing the best [27].

#### Recombination

To generate the chromosome of the offspring, we recombined the chromosomes of their parents by choosing a random position in the chromosome. Then, the chromosome of each offspring was obtained by applying one-point crossover used in evolutionary computing [27].

Before performing recombination, we checked whether either the male or female was infected with *Wolbachia*. As described in the *Wolbachia* section, if the male is infected and the female not, all the offspring are killed by the cytoplasmic incompatibility. In this case, there is no need to generate the chromosome of the offspring. To keep the size of the population constant, the mating process continues until all the slots in the population are completely filled.

If offspring are feasible, recombination is performed with a probability of 100 %. Recombination is always performed because in nature, the mosquito offspring always inherit genetic material from both parents. Recombination does not directly affect the results of the simulation, but it is important for genetic evolution analyses to be performed in the future.

After recombination is performed, the *Wolbachia* status of the offspring is set depending on the parents’ infection status, as explained previously.

#### Mutation

In all the experiments presented in this paper, we employed the mechanism of uniform mutation borrowed from the evolutionary computation literature [27]. For each position in the chromosome, we generated a random number; if it surpassed a certain threshold, the chromosome was mutated at that position.

For each position in the chromosome that needed to be mutated, we generated another random number. Based on this generated number, another letter of the DNA alphabet was selected from a uniform probability distribution to replace the mutated letter.

As with recombination, mutation does not directly affect the results of the simulations, but both are necessary to produce data on genetic change in populations for genetic evolution analyses.

#### Migration

Migration is the process whereby some individuals are relocated in other nearby populations. This mechanism is very important in nature because it introduces genetic diversity in populations. In our experiments, migration was simulated by moving a proportion of individuals from one population to another. This process occurred before each generation was created.

Newly migrated individuals participate in the reproduction process, introducing genetic diversity into the population and in some cases, affecting the degree of *Wolbachia* invasion in the population.

## Results

In this section, we present a series of experiments that allow us to outline different simulations that can be conducted using the computer model and the questions that can be addressed with these experiments.

The parameters used in the experiments are divided into two groups. The first group contains the control variables that are fixed in all the experiments. After running several experiments, we observed that the effect of these parameters on the outcome of the simulations is marginal. The first group of parameters with their respective values are given in Table 1.

**Table 1:** Fixed parameters. Maximum Offspring refers to the maximum number of offspring reaching adulthood from a single female

Parameter	Value
Chromosome Length	200 bases
Generations	100
Mutation Probability	1 %
Possible Mating Partners	3
Maximum Offspring	10
Neighborhood Size	30
Recombination Probability	100 %

The second group consists of those parameters that are most important in terms of the goals of these experiments. Moreover, in field experiments with real mosquitoes, these parameters comprise the independent variables that can be manipulated. To obtain the values for these parameters, we used a statistical method known as Latin Hypercube Sampling (LHS), which is frequently used to construct computer experiments. LHS generates a plausible set of parameter values from a multidimensional distribution [28]. To determine the ranges of these parameters, we used information from the literature [29, 30].

Table 2 lists these parameters and their values, represented as a range, which differs from the single values given for each of the parameters included in group 1.

**Table 2:** Variable *Wolbachia* and mosquito parameters. Cytoplasmic Incompatibility, Maternal Inheritance, and Fecundity Penalty were obtained from the wMel estimates in [7]. Fitness Cost was obtained from [5]. Mosquito and *Wolbachia* variables are denoted by (M) and (W), respectively

Parameter	Value
Population Size (M)	10,000–250,000
Number of Populations (M)	2–4
Invasion Rate (M)	20–40 %
Migration (M)	1–5 %
Intensity of Cytoplasmic Incompatibility (W)	100 %
Maternal Inheritance (W)	100 %
Fecundity Penalty *Wolbachia* Infected (W)	12–18 %
Fecundity Penalty *Wolbachia* Non-infected (W)	8–10 %
*Wolbachia* Fitness Cost (W)	15–25 %

We conducted several experiments with different combinations of parameter values to simulate the most relevant scenarios. Owing to the large number of combinations of parameters values, we gathered a large volume of data from which we selected, analyzed, and synthesized the most important results, as described below.

In all experiments, at least 30 independent runs were performed to ensure that the results obtained would be statistically valid. The results of the experiments are presented as averages of all runs. We conducted five different experiments, in each of which, the values of one important parameter were varied while the others remained fixed. The experiments were separated because we wished to observe the effect of each parameter on the invasion process.

The experiments were designed based on mathematical models of *Wolbachia* invasion [9, 10]. We also considered the results from the Eliminate Dengue empirical study in which *Wolbachia* infected mosquitoes were released and then recaptured to measure the degree of invasion of infected mosquitoes within the native populations [5].

**Fig. 3:**
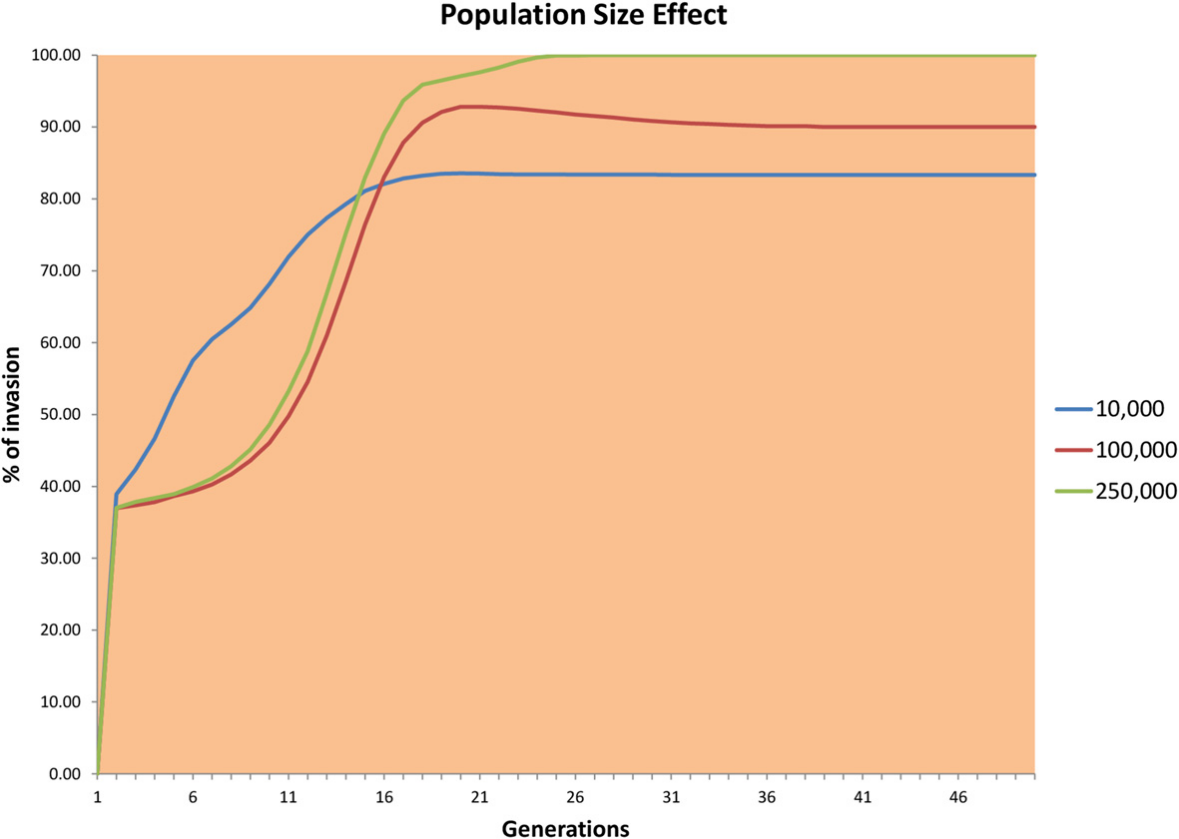
Invasion using different *Wolbachia* fitness costs averaged over 30 runs. The lines represent the invasion rates using different introduction rates of infected mosquitoes

The general algorithm applicable to all the experiments is described in Algorithm 1:



The results of this experiment are shown in Fig. 3.

**Fig. 4:**
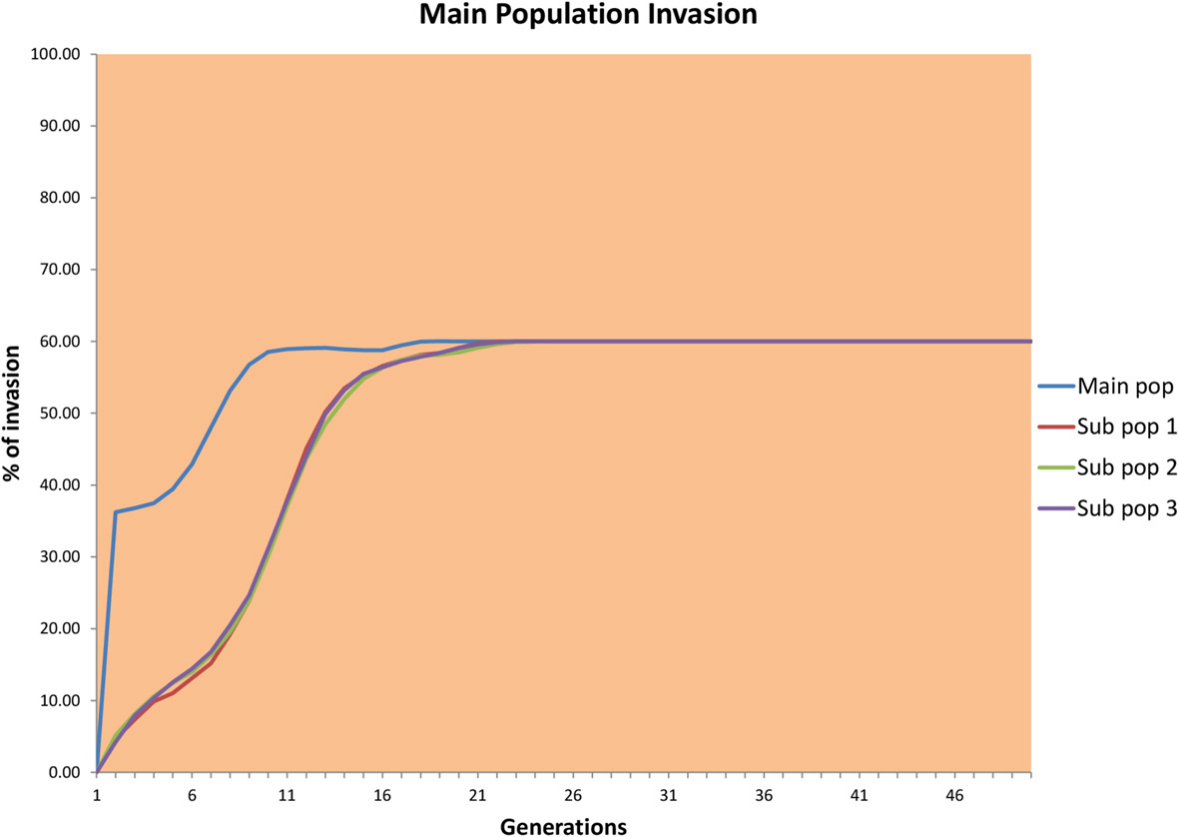
General model used in the experiments

A general pictographic representation of how the experiments were conducted is shown in Fig. 4.

### Infection rate

The goal of this experiment was to find the threshold for the initial *Wolbachia*-infected population size necessary to invade the native population fully. We investigated three different fitness costs due to *Wolbachia* infection: 15 %, 20 %, and 25 %. Moreover, different rates of initial invasion were used to find the unstable equilibrium point, that is, to detect the thresholds where the infection completely disappears or spreads among all or almost all the individuals. We used large populations of 90,000 individuals because we needed a population that was sufficiently large to be refractory to genetic drift and, although rare, some main populations could reach that size [29]. The main population is the source population in the area and is generally close to a human village and a water body, providing food and a hatching site, respectively.

The results of this experiment, shown in Fig. 3, are consistent with the mathematical models. In each case, the infection spreads or decreases rapidly at the beginning and after about 15 generations remains almost unchanged. The most valuable information obtained from this experiment is the equilibrium points and thresholds for different *Wolbachia* fitness costs and invasion rates.

In the past, we tested other population modification alternatives using computer simulations. In our experiments, *Wolbachia* infection was proved to be superior in terms of the percentage of infected mosquitoes needed for *Wolbachia* to invade the native population [13, 15].

### Population size effect

**Fig. 5:**
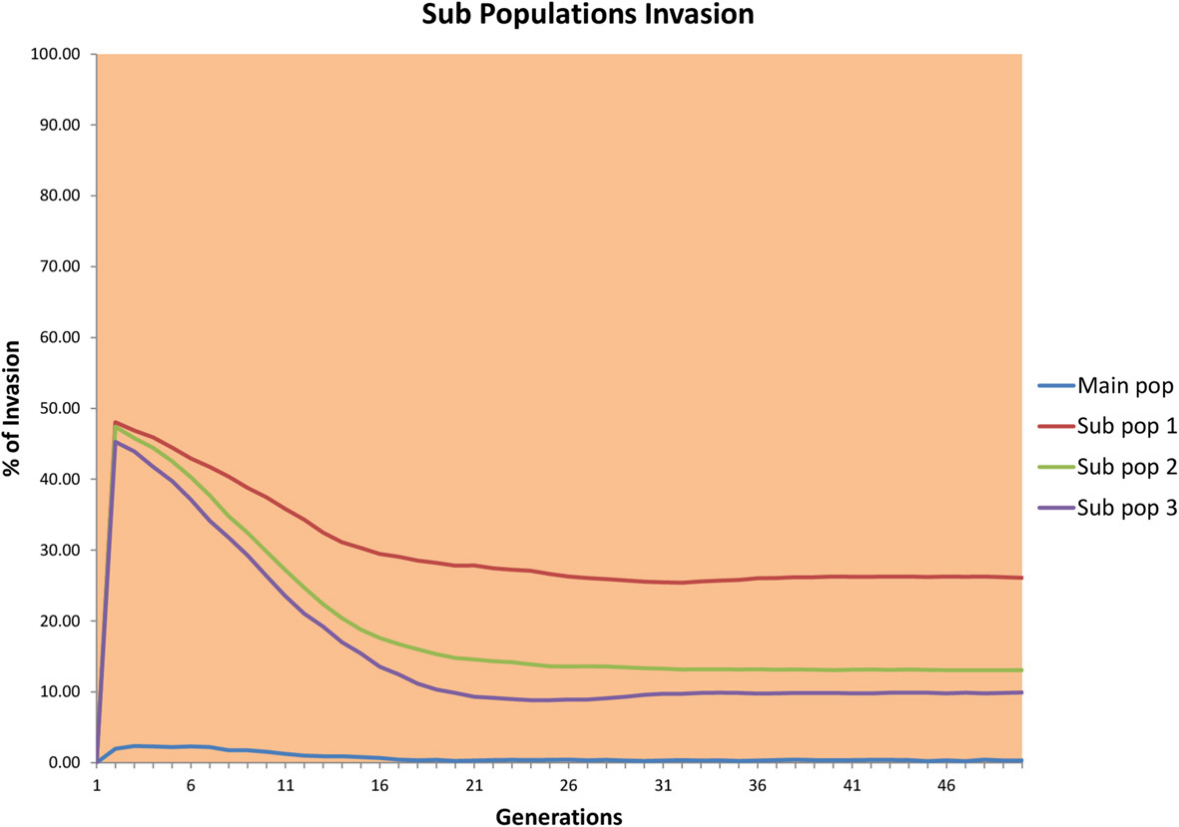
Effect of population size using 10,000, 100,000, and 250,000 individuals

The aim of this experiment was to observe the effect of population size on the spread of *Wolbachia* infection. In this experiment, the initial invasion rate was fixed at 30 % and the number of individuals in each population varied over the range given in Table 2. Figure 5 shows the results of the experiment.

Figure 5 shows that in larger populations, *Wolbachia* infection spreads much faster. In the largest population, all the individuals were infected within only 25 generations, whereas in the smaller population, the infection rate remained between 80 % and 93 %. This behavior results from the influence of genetic drift, which induces erratic population dynamics.

It should be noted that in most of the simulations with the smallest population (10,000 individuals) and several of the simulations with the medium size population (100,000), the infected population became extinct in several runs, thereby lowering the average invasion rate in these cases.

The outcomes of these experiments suggest that it is easier and faster to invade a large population completely than to invade a small population even with the introduction of a larger percentage of *Wolbachia* infected mosquitoes.

### Number of populations

In many natural environments, there are more than one mosquito population in a geographic area connected via migration. In this experiment, our goal was to observe the effect of the number of mosquito populations on the *Wolbachia* invasion process and how it spreads among several populations. We simulated three and four populations of equal size connected via migration and compared the results of the two scenarios. A source population and two or three sub populations around the source were used without any migration between sub populations. In each population, a percentage of infected mosquitoes was introduced at the beginning of the experiment according to Table 2.

The results of this experiment are shown in Table 3.

**Table 3:** Pearson’s correlation coefficients

	Population 1	Population 2	Population 3	Population 4
Population 1	1.0000000	0.9456096	0.9424586	0.9105121
Population 2		1.0000000	0.9957714	0.9904861
Population 3			1.0000000	0.9896529
Population 4				1.0000000

We computed Pearson’s correlation coefficients for all pairs of experiments (see Table 3). High correlation coefficients indicate that the number of populations does not have a significant impact on the spread of the infection. As the number of populations increases, there is a tiny perturbation on how the infection spreads among the populations owing to migration, but it is not important enough for the number of populations to be considered a crucial factor.

### Island model scenario

Using the results of the previous experiments, we designed an additional experiment that follows the island model. In this experiment, there were four populations, one of which was the source population while the other three were sub populations connected via migration. There was no migration between sub populations. The goal of this experiment was to compare two different scenarios. In the first scenario, *Wolbachia* infected mosquitoes were introduced into the source population, while in the second, they were introduced only into all the peripheral populations, each of which comprised 1,000 individuals. Parameters differing from those used in the other experiments are shown in Table 4.

**Table 4:** Simulation parameters. For both scenarios, the number of *Wolbachia* infected mosquitoes inserted were fixed at 3,000 mosquitoes representing 30 % of the main population

Parameter	Value
Main Population Migration	5 %
Peripheral Population Migration	3 %
Main Population Size	10,000
Peripheral Population Size	2,500
*Wolbachia* Infected Mosquitoes Inserted	3,000

**Fig. 6:**
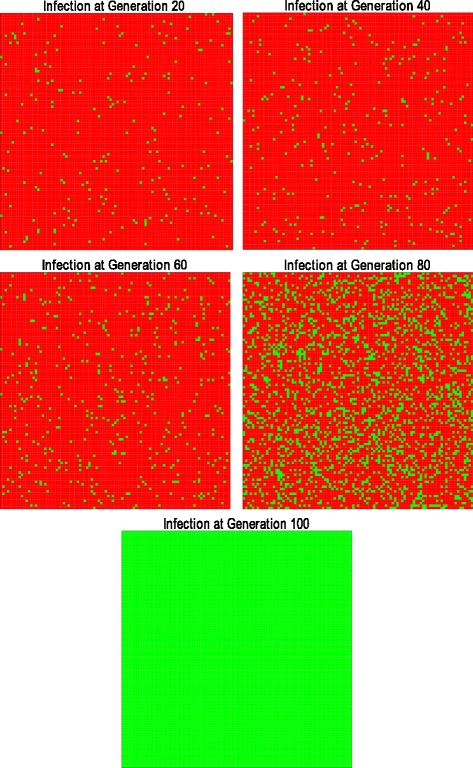
Infection of source population

The results of the first scenario are shown in Fig. 6.

Figure 6 shows that for all populations, approximately 60 % invasion was achieved by the end of the simulation. The degree of invasion was a little lower than that obtained with a single population owing to migration, which lowers the invasion in the main population. We used smaller populations because as found in a previous experiment, small populations are harder to infect and in nature, population sizes as small as 10,000 individuals are common [29].

**Fig. 7:**
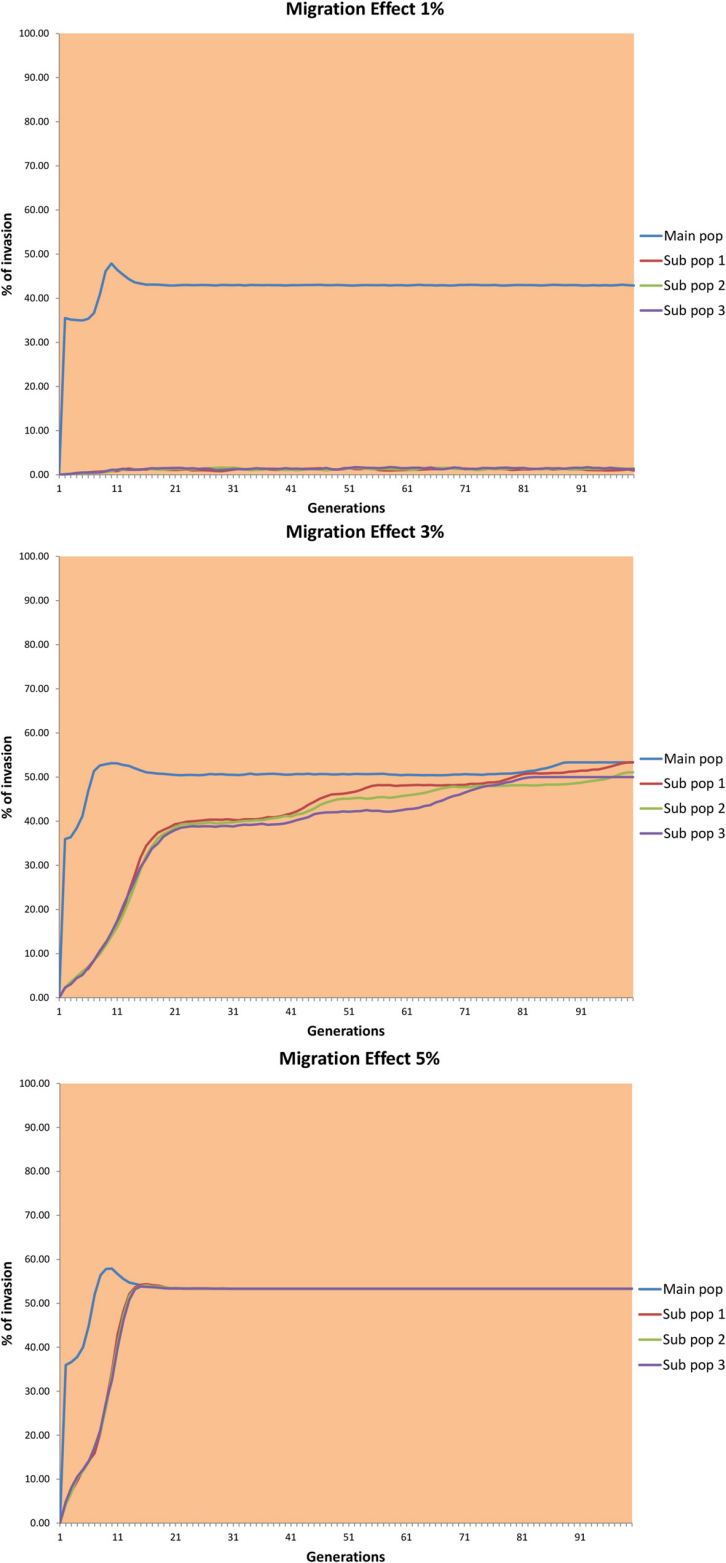
Infection of peripheral populations

Figure 7 shows how the invasion of *Wolbachia* in the subpopulations starts at an accelerated rate but becomes, after a few generations, constant and then finally starts to decrease. In the main population, the fixation of *Wolbachia* starts very slowly and remains almost constantly close to zero. Neither the main nor the subpopulations could be invaded.

#### Main population infection spread

**Fig. 8:**
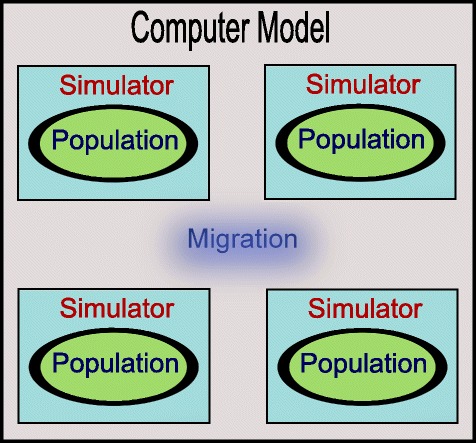
Spread of infection through the generations.In this example, the infection reached 100 % of the individuals of the population

Figure 8 shows how the infection spreads among the native population through the generations in the scenario where the infected mosquitoes are introduced into the main population, leaving the sub populations unaltered.

In the first few generations, the infection remains almost constant until it reaches approximately 35 % invasion in the fourth generation. Once this threshold is reached, the infection spreads very rapidly until all the individuals reach the equilibrium point by generation 20.

Figure 8 also shows that the infection spreads evenly among the population. There are no areas in which the infection is more concentrated or has little presence.

### Effect

Migration is a critical factor for spreading the *Wolbachia* infection in the island model scenario [30]. The goal of this experiment was to observe how different migration rates affect the spread of *Wolbachia* bacteria among the source population and subpopulations. It is believed that several populations can coexist with stable rates of *Wolbachia* infection if they are connected via migration. To conduct these experiments we used the same scenario as in the previous experiment (Island Model Scenario), infecting only the source population and using three different migration rates from the main population to the sub populations, namely, 1 %, 3 %, and 5 %. The rest of the parameters used are listed in Table 4.

**Fig. 9:**
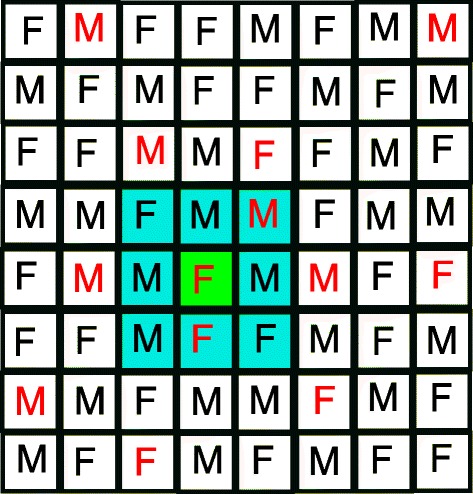
Results using 1 %, 3 %, and 5 % migration between source population and peripheral populations

Figure 9 shows the results of these experiments. In the first scenario, the degree of invasion of the source population increases rapidly until it reaches the equilibrium point in generation 15. Invasion of the subpopulations remains close to zero owing to the low migration rate, which hinders the infected mosquitoes from migrating to the subpopulations in sufficient quantities to invade them consistently.

In the other two scenarios, all the populations reached a stable and almost identical *Wolbachia* degree of invasion. Moreover, the higher the migration rate was, the faster the populations reached equilibrium with an approximate 53 % invasion. Apparently, all populations reached equilibrium when using a 3 % or higher migration rate.

Specifically, if the migration rate is too high, it can happen that most of the infected mosquitoes leave the source population and settle in a sub population, leading to rapid invasion of the sub populations, but leaving the source population with too few infected individuals to spread the bacteria. Conversely, if the migration rate is too low, the source population can be infected rapidly but the sub populations remain uninfected or with a very low degree of invasion because very few mosquitoes reach the sub populations.

These results confirm that migration is a very important factor in the spread of the *Wolbachia* bacteria to other populations and in maintaining the degree of *Wolbachia* invasion at a stable level [30, 31].

## Conclusions

The work presented here shows that computer modeling and simulations are useful tools for studying the dynamics of *Wolbachia* invasion. We showed that computational simulations can provide important insight into the conditions required to implement biological strategies for controlling vector-borne diseases such as malaria and Dengue.

Elucidation of these conditions experimentally would be onerous in practice. We believe that computational simulations are useful tools for modeling population-based phenomena, such as evolution, migration, and bacterial invasion, among others. Furthermore, computational modeling can be used to validate mathematical population models and is an excellent low-cost alternative to experimental studies.

In particular, our computer simulations suggest that *Wolbachia* invasion can be rapidly achievxed in simulated populations under certain doable conditions. The results obtained in our experiments are consistent with the observations derived from both mathematical model predictions and field experiments on *Wolbachia* transmission in real mosquito populations [5, 7–10]. This implies that the cost of experimental studies can be reduced by conducting computer simulations to predict the spread of *Wolbachia* invasion in advance. Moreover, we believe that these predictive studies have great potential in contributing to the efficacy of *Wolbachia* invasion experiments aimed to reduce the spread of Dengue.

Computer simulations, however, have obvious limitations. The dynamics of infectious diseases is a complex multifactorial phenomenon. Estimation of the factors influencing the invasion rate and the modeling thereof seems intractable from a computational perspective. Therefore, assessing the effectiveness of computer simulation models and parameterizing them with real data could provide useful insight into the capabilities and limitations of computer simulations, providing the basis for improving their prediction capabilities.

Consequently, our future work will focus on assessing the results of the proposed model by conducting statistical analyses and comparing the results in the light of new data made available from real world experiments on *Wolbachia* invasion. Overall, we believe that this work could contribute to the eventual deployment of biological strategies for controlling vector-borne diseases generally.

The source code and binary executable of the software are available on request from the corresponding author.
